# Chromosomal abnormalities related to fever of unknown origin in a Chinese pediatric cohort and literature review

**DOI:** 10.1186/s13023-022-02444-0

**Published:** 2022-07-27

**Authors:** Bijun Sun, Mi Yang, Jia Hou, Wenjie Wang, Wenjing Ying, Xiaoying Hui, Qinhua Zhou, Haili Yao, Jinqiao Sun, Xiaochuan Wang

**Affiliations:** 1 Department of Clinical Immunology, Children’s Hospital of Fudan University, 399 Wanyuan Road, Shanghai, 201102 China; 2 Department of Pediatric, Xiamen Children’s Hospital, Xiamen, 361000 China; 3 Shanghai Institute of Infectious Disease and Biosecurity, Shanghai, 200032 China

**Keywords:** Fever of unknown origin, Chromosomal abnormalities, Copy number variations, Autoinflammatory, Central fever

## Abstract

**Background:**

Fever of unknown origin (FUO) has been difficult to diagnose in pediatric clinical practice. With the gradual change in the disease spectrum, genetic factors have received increasing attention. Limited studies have shown an association between FUO and chromosomal abnormalities. In this study, we investigated the clinical and genetic characteristics of patients with FUO presenting with chromosomal abnormalities in a Chinese pediatric cohort.

**Results:**

Chromosomal abnormalities were detected in 5.5% (8/145) of the patients with FUO. Six patients with inflammatory fever presented with pharyngitis/amygdalitis (4/6), oral aphthous ulcer (2/6), digestive symptoms (3/6), developmental delay (4/6) and elevated C-reactive protein levels (6/6) during fever. These patients were often considered to have systemic inflammatory diseases, such as Behcet’s disease or systemic juvenile idiopathic arthritis. Trisomy 8, 7q11.23 dup, 3p26.3-p26.1 del/17q12 dup, 22q11.21 del, and 6q23.3-q24.1 del were identified in patients with inflammatory fever. The *TNFAIP3* gene was included in the 6q23.3-q24.1 deletion fragment. Two patients with central fever were characterized by facial anomalies, developmental delay, seizures and no response to antipyretic drugs and were identified as carrying the de novo 18q22.3-q23 del. By performing a literature review, an additional 19 patients who had FUO and chromosomal abnormalities were identified. Trisomy 8, 6q23.2-q24.3 del and 18q22.3-q23 del were reported to present as fever, similar to the findings of our study.

**Conclusions:**

We emphasized the important role of detecting chromosomal abnormalities in patients with FUO, especially in patients with systemic inflammatory manifestations or developmental delay. Identifying chromosomal abnormalities may change the diagnosis and management of patients with FUO.

## Background

Fever is a common manifestation of various diseases in children. Fever of unknown origin (FUO) has also been particularly difficult to specifically diagnose in pediatric clinical practice since Petersdorf and Beeson first defined it in 1961 [1]. Previous reports suggested that 40%-50% of pediatric patients with FUO were identified as having an infection. Noninfectious etiologies, including autoimmune disease, malignancy, environmental stressors or drugs, also play important roles in FUO diagnosis [2]. However, the causes of fever in some children are still unexplained.

Autoinflammatory and thermic dysregulation may be potential causes of FUO. Autoinflammatory diseases mainly present as early-onset recurrent fever and systemic inflammation and involve various organs and tissues, such as the skin, mucosa, joints, gastrointestinal tract, and central nervous system. This type of disease is characterized by dysregulation of the innate immune system with antigen-independent immune pathway hyperactivation and the development of sterile inflammation [3, 4]. After the identification of the first gene, *MEFV*, which results in familial Mediterranean fever [5], more than 50 new monogenic autoinflammatory diseases have been discovered due to advances in genetic sequencing and are known as important causes of unexplained fever in children. Central fever is caused by abnormalities in the thermoregulatory center and therefore an elevated body temperature range. The outstanding characteristics of central fever are a lack of infectious symptoms and lack of response to antipyretic treatment. An abnormal thermoregulatory center may result from craniocerebral diseases, such as cerebral hemorrhage, traumatic brain injury, and brain surgery [6]. In pediatric patients, central fever often occurs with the following diseases: cerebral dysplasia, seizures and developmental delay [7], which often have significant genetic factors.

Despite remarkable progress in understanding the pathogenesis of FUO, its accurate diagnosis and management remain very difficult. Previous studies have mainly focused on the clinical etiology of FUO rather than genetic factors. In addition to single genes, genetic factors include chromosomal abnormalities. Chromosomal abnormalities are further categorized as numerical or structural. Copy number variation (CNV) is a type of chromosomal deletion or duplication variation ranging in size from 1 kb to several Mb. Chromosomal abnormalities, especially CNVs, have been indicated to be linked to a number of diseases, such as neurological disorders and congenital malformations [8]. In a few studies, CNVs have been recognized as an important cause of inflammatory fever [9]. Recent discoveries suggest that pathogenic CNVs using next-generation sequencing (NGS)-based methods have provided genetic characteristics of many previously undiagnosed patients with systemic autoinflammatory diseases, especially if single genes have not been confirmed [10].

However, the specific proportions and characteristics of CNVs in patients with FUO remain unclear and should not be ignored by clinicians. In this study, patients with FUO who were treated at a single center in China were enrolled, their genetic backgrounds were analyzed, especially for chromosomal abnormalities, and the relationship between chromosomal abnormalities and fever was explored to improve our understanding of the influence of genetics on FUO.

## Methods

This study was approved by the Ethics Committee of Human Experimentation in Children’s Hospital of Fudan University. Written informed consent was obtained from the parents of all patients.

### Patients

Children with fever of unknown origin (FUO) and recurrent fever who were admitted to our hospital between January 2016 and June 2021 were included in this study. We excluded patients who were clinically diagnosed with infections, connective tissue disease and malignant tumors. FUO was defined as an elevated temperature (> 38.3℃ measured by the rectal route or > 37.3℃ by the axillary route) for more than 3 weeks or a failure to determine a diagnosis after 1 week of inpatient investigations [11]. Recurrent fever was defined as at least three episodes of unexplained fever in a six-month period, with a minimum interval of seven days between episodes [12, 13]. The relevant data are summarized in detail. Data from patients with FUO who had chromosomal abnormalities and were previously reported in PubMed Medline (https://www.ncbi.nlm.nih.gov/pubmed/) were reviewed.

### Genomic DNA extraction

Genomic DNA was extracted from peripheral blood samples collected from patients and their parents using a TINGEN genomic DNA purification kit (TINGEN, Tianjin, China) according to the manufacturer’s instructions. The DNA concentration and absorbance were determined using a NanoDrop spectrophotometer (NanoDrop Technologies, Berlin, Germany).

### Next-generation sequencing

Genomic DNA fragments were enriched using the Agilent SureSelect XT Human All Exon V5 kit. After enrichment, DNA libraries were sequenced with the HiSeq 2000 platform according to the manufacturer’s instructions (Illumina, San Diego, CA) with an average on-target sequencing depth of 120 × . More information about sequencing and data analysis, particularly of single nucleotide variations, can be found in a previous study [14]. The pipeline for clinical NGS-involved CNV detection (PICNIC) was used to detect CNVs from whole-exome sequencing (WES) data. PICNIC filters out high-frequency gene deletions/duplications. The detailed process for CNV analysis was presented in published studies [15, 16]. CNVs were further verified by performing comparative genomic hybridization (CGH) or multiplex ligation-dependent probe amplification (MLPA).

### Array CGH

We applied an Agilent custom Human Genome CGH microarray 4 × 180 K (Agilent Technologies, Santa Clara, CA, USA) for CNV analysis. Array CGH experiments were performed using standard protocols provided by the manufacturer. The analysis was performed using Agilent CytoGenomics software (Agilent Technologies, version 2.5.8.11) [15].

### MLPA

An MLPA probe set (MRC Holland, P029-C1) was used to detect chromosomal imbalances. This kit contained probes of genes mapped to the 7q11.23 critical region (*CLIP2, ELN,* and *LIMK1*). MLPA was carried out according to the standard protocol (MRC Holland, MLPA® General Protocol MDP-v007, Amsterdam, Netherlands). The electrophoresis was performed using an ABI 3130 Genetic Analyzer, and data were analyzed by GeneMarker software (SoftGenetics, State College, United States, vision 1.95). The results were considered abnormal when the relative peak height ratio was below 0.7 or above 1.30.

## Results

### Chromosomal abnormalities in FUO

**Fig. 1:**
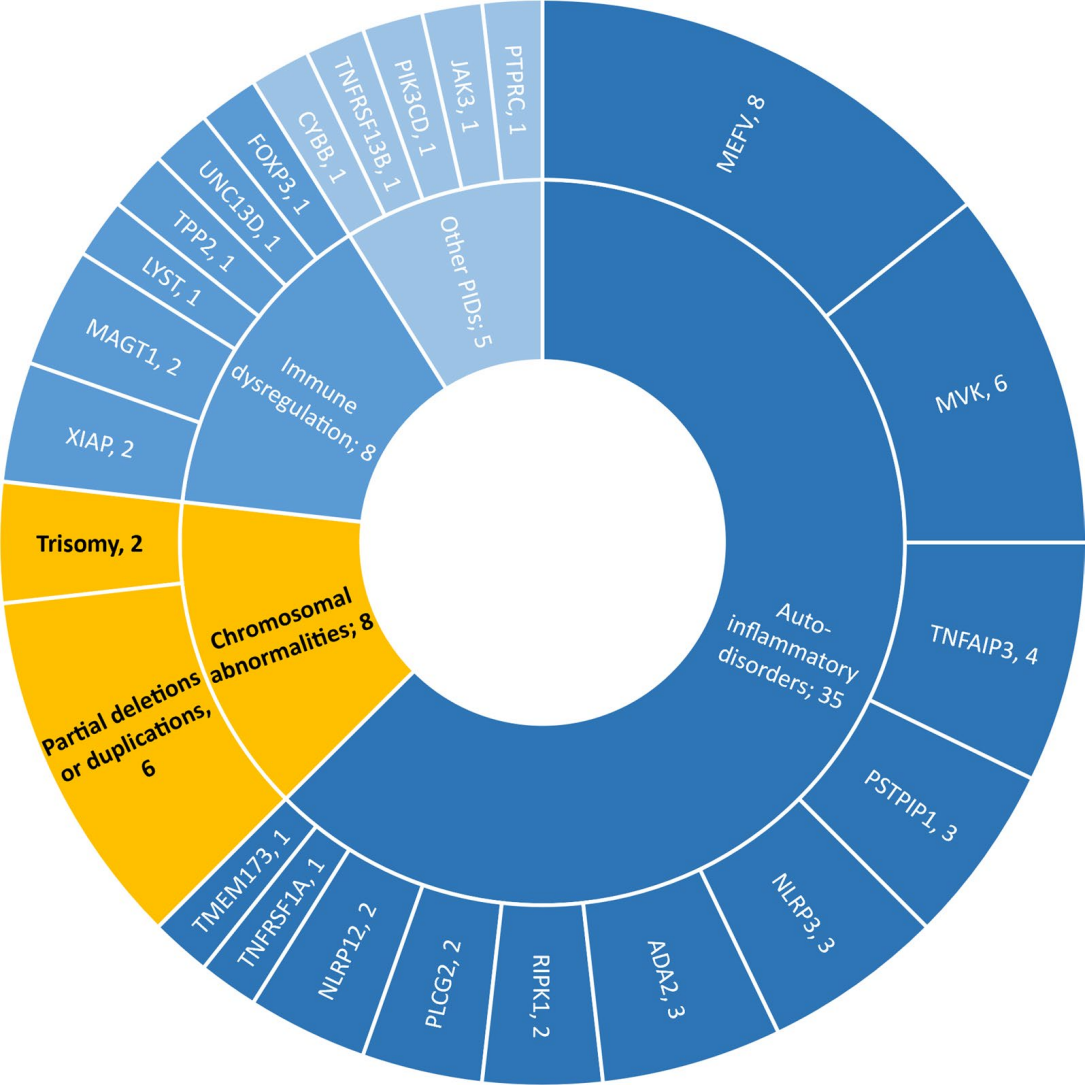
FUO with genetic abnormalities in our cohort. Chromosomal abnormalities are marked in yellow

One hundred forty-five patients with FUO or recurrent fever were enrolled in our cohort, and trio-WES was performed. Ultimately, 33.1% (48/145) of patients had single gene mutations of primary immunodeficiency diseases. Notably, in addition to monogenic diseases, 8 patients (P1-P8) had chromosomal abnormalities, which accounted for 5.5% (8/145) of the total patients with FUO in our cohort (Fig. 1). The characteristics of these 8 children (P1-P8) with chromosomal abnormalities are further summarized in Table 1. The patients with chromosomal abnormalities were further divided into 6 patients with inflammatory fever and 2 patients with central fever.

**Table 1:** Baseline characteristics of FUO patients with chromosomal abnormalities

Patient	P1	P2	P3	P4	P5	P6	P7	P8
Fever phenotype	Periodic fever	Periodic fever	Periodic fever	Periodic fever	Periodic fever	Recurrent fever	Central fever	Central fever
Age at fever presentation	10 m	36 m	8 m	10 m	24 m	2 m	9 m	6 m
Age at diagnosis	56 m	67 m	21 m	44 m	60 m	45 m	22 m	14 m
Gender	F	F	M	F	F	F	M	M
Fever interval	1 m	1 m	1 m	0.5–1 m	2 m	irregular	persistent	persistent
Facial abnormality	–	–	–	–	–	frontal bossing	+	+
Congenital anomalies	VSD/ASD	–	–	flexion deformity of finger	–	PDA	+	congenital aural atresia
Growth retardation	–	–	+	–	–	+	+	+
Developmental delay	+	+	+	–	–	+	+	+
Pharyngitis/ amygdalitis	+	+	+	+	–	–	–	–
Lymphadenectasis/ hepatosplenomegaly	–	–	+	+	+	+	–	–
Skin/mucous	–	–	–	Oral aphthous perianal abscess	Oral aphthous	rash	–	–
Gastrointestinal	Vomiting	–	–	Diarrhea	Abdominal pain	–	–	–
Arthritis	–	–	–	–	–	+	–	–
Blood	–	–	–	–	anemia, thrombocytopenia	HLH	–	–
Central nervous system	Tourette syndrome	–	–	Seizure	–	Seizures	Seizures	Hypotonia; abnormal EEG
Abnormal chromosome and gene	7q11.23 dup	3p26.3–p26.1 del;17q12 dup	22q11.21 del	Trisomy 8	Trisomy 8; *PTPN11*: c.1508G > C	6q23.3–q24.1 del	13q32.3–q34 dup;18q22.3–q23 del	18q22.3–q23 del
RefSeq genes included in abnormal chromosome	*NCF1, LAT2, PTPN12, MERM1*	*TRNT1, CCL5, CCL2, CCL16,* etc	*SCARF2,*etc	*IKBKB, TNFRSF11B,* etc	*IKBKB, TNFRSF11B,* etc	*TNFAIP3, IFNGR1,* etc	*TNFSF13B, GALR1, TSHZ1,* etc	*GALR1, TSHZ1,* etc
Treatment	Antibiotic, colchicine, bacterial lysates	Antibiotic, temporary corticosteroid, bacterial lysates	Antibiotic, single–dose corticosteroids during attacks	Antibiotic, temporary corticosteroid	Antibiotic, mesalazine, bacterial lysates	Antibiotic, long–term corticosteroids, NSAIDs	Antibiotic, thymopolypeptides, antiepileptic	Antibiotic, rehabilitation training

*m* months; *F* female; *M* male; *VSD* ventricular septal defect; *ASD* atrial septal defect; *PDA* patent ductus arteriosus; *HLH* hemophagocytic lymphohistiocytosis; *EEG* electroencephalogram; *NSAIDs* nonsteroidal anti-inflammatory drugs

### Clinical manifestations of patients with inflammatory fever

Six patients (P1-P6) presented with inflammatory fever, with a median onset age of 10 months (range, 2 months-36 months). Five (P1-P5) of them had periodic fever, lasting for 3–7 days and usually recurring at intervals of 1–2 months, while one patient (P6) had intermittent fever with a recurrence frequency of 7–8 times per year. These patients suffered from hyperpyrexia with a temperature spike > 38.5 °C (axillary temperature).

Abnormal inflammation in other organs during fever was prominent. Pharyngitis and tonsillitis were the most frequent concomitant symptoms, which occurred in 4/6 patients (P1-P4). The affected skin and mucosa were characterized by recurrent oral aphthous ulcers (P4 and P5) and red maculopapules during fever (P6). Digestive symptoms included vomiting, chronic diarrhea and abdominal pain. Moreover, a thickened terminal ileum wall was discovered in P4 by abdominal CT, and perianal, colon and intestinal ulcers were detected in P5 using enteroscopy. Four patients had lymphadenectasis (P3-P6), three of whom had hepatosplenomegaly (P4-P6). Two patients had hematological involvement during fever. P5 presented with moderate anemia and thrombocytopenia that was able to rise to normal after temperature control, and the peripheral blood smear was normal at the beginning of the disease. She subsequently had persistent thrombocytopenia and a progressive decline in hemoglobin and was eventually diagnosed with acute myeloid leukemia (AML) at 69 months of age. Moreover, P6 was suspected to have hemophagocytic lymphohistiocytosis (HLH) at the age of 2 years because of pancytopenia, and her blood cell counts increased after corticosteroid therapy. In addition to the inflammatory manifestations, four patients (P1-P3 and P6) had developmental delay in motor or language, and two of them (P3 and P6) also presented growth retardation.

During fever attacks, the levels of inflammatory markers were markedly increased (Table 2). Elevated white blood cell count (WBC) (5/6), C-reactive protein (CRP) (6/6), erythrocyte sedimentation rate (ESR) (6/6) and serum amyloid A (SAA) (2/2) were detected. The acute phase reactants plummeted to normal levels when the body temperature stabilized. Routine immunological function tests, in comparison with the reference values in healthy children in China [17], showed that T lymphocytes decreased and immunoglobulin E (IgE) levels increased significantly in P5, and the CD4/CD8 ratio of P6 was reversed. The etiological examinations, such as blood culture, urine culture, and throat swab culture, were all negative. The possibility of *Mycobacterium tuberculosis*, Epstein–Barr virus, and cytomegalovirus infection was excluded after pathogen antibody and DNA detection.

**Table 2:** Immunophenotypes and inflammatory markers of FUO patients with chromosomal abnormalities in our cohort

Patient	P1	P2	P3	P4	P5	P6	P7	P8	Reference range
Age	52 m	67 m	21 m	48 m	60 m	51 m	22 m	12 m	
Gender	F	F	M	F	F	F	M	M	
WBC (cells/ul)	23,300↑	5700	23,210↑	23,400 ↑	18,000↑	15,700↑	8300	10,700	4000–12,000
NEUT (%)	71.4↑	55	74.4↑	55	85.4↑	57	59.2	25.8	30–70
CD19 (%)	25.35↑	15.38	24.38	18.2	9.31↓	19.05	35.7↑	18.82	12-24 m: 13.23–26.39; 48-72 m: 10.46–21.77
CD19 (cells/ul)	788.55↑	483.06	1475.59↑	495.95	142.81↓	678.93	1006.77	917.87	12-24 m: 461–1456; 48-72 m: 303.52–777.25
CD3 (%)	65.62	74.35	63.69	66.63	69.94	67.36	45.38↓	61.32	12-24 m: 53.88–72.87; 48-72 m: 59.50–75.56
CD3 (cells/ul)	2041.2	2335	3854.4	1815.9	1072.6↓	2400.8	1279.8↓	2990	12-24 m: 1794–4247; 48-72 m: 1480–2847
CD4 (%)	38.47	42.00↑	38.26	38.69	53.14↑	17.62↓	31.03	36.82	12-24 m: 24.08–42.52; 48-72 m: 28.49–41.07
CD4 (cells/ul)	1196.79	1319.18	2315.53↑	1054.46	814.94	628.14↓	875.25↓	1795.57	12-24 m: 902–2253; 48-72 m: 767–1592
CD8 (%)	21.96	25.5	21.74	21.95	16.3↓	46.56↑	10.08↓	22.17	12-24 m:19.00–32.51; 48-72 m: 19.70–32.04
CD8 (cells/ul)	683.00	800.81	1315.9	598.19	249.91↓	1659.24↑	284.28↓	1080.9	12-24 m:580–1735; 48-72 m: 553–1127
CD16CD56 (%)	6.74↓	8.84	11.24	13.61	18.72	12.83	16.54	18.65	12-24 m: 7.21–20.9; 48-72 m: 7.83–20.99
CD16CD56 (cells/ul)	209.56↓	277.64	680.25	370.2	287.17	457.14	466.52	909.37	12-24 m: 270–1053; 48-72 m: 227–668
IgG (g/L)	7.60	13.2 ↑	7	10.5	19.8 ↑	6.5	7.1	10.2↑	12-24 m: 5.52–11.46; 48-72 m: 4.95–12.74
IgM (g/L)	1.55	2.27 ↑	1.01	1.81	1.96	1.59	0.7	0.37	12-24 m: 0.6–2.12; 48-72 m: 0.65–2.01
IgA (g/L)	0.52	1.19	0.48	0.67	2.8 ↑	0.85	0.3	0.21	12-24 m: 0.06–0.74; 48-72 m: 0.33–1.89
IgE (KU/L)	171.6 ↑	32.55	34.18	31.13	3006.67 ↑	37.42	7.1	29.72	< 100
CRP (mg/L)	87 ↑	16 ↑	27.18 ↑	70 ↑	40 ↑	132 ↑	< 8	< 0.5	< 8
ESR (mm/h)	48 ↑	32 ↑	64 ↑	44 ↑	54 ↑	107 ↑	35↑	2	0–26
SAA (mg/L)	NA	259.6 ↑	256.4 ↑	NA	NA	NA	< 2.5	< 2.5	< 10
IL-2 (pg/ml)	NA	1.3*	0*	NA	1.71	NA	0	2.3	0–11.4
IL-4 (pg/ml)	NA	1.35*	0*	NA	2.32	NA	0	0.8	0–12.9
IL-6 (pg/ml)	109.6 ↑	2.86*	0.8*	7.6*	44.79 ↑	667.7 ↑	0	5.9	0–16.6
IL-8 (pg/ml)	NA	NA	NA	NA	NA	4956.86 ↑	NA	NA	0–21.4
IL-10 (pg/ml)	NA	3.56*	2.1*	NA	10.64 ↑	12.95 ↑	1.07	6	0–5.9
TNF-α (pg/ml)	NA	2.27*	0*	NA	90.96 ↑	11.03↑	0	3.6	0–5.5
IFN-γ (pg/ml)	NA	2.64*	0*	NA	NA	NA	0	1.5	0–17.3

*m* months; *F* female; *M* male; *WBC* white blood cells; *NEUT* Neutrophil count; *CRP* C-reactive protein; *ESR* erythrocyte sedimentation rate; *SAA* Serum amyloid A; *IL* interleukin; *TNF* tumor necrosis factor; *IFN* interferon; *NA* not available

The percentage and numbers of lymphocyte subsets in the peripheral blood reference to the literature [17]

*Without fever

Therefore, several inflammatory diseases were initially considered, and anti-inflammatory therapy was given to these patients due to their inflammatory manifestations. For example, P3 had periodic fever, tonsil exudation, cervical lymph node enlargement, and increased inflammatory indicators, which were consistent with the main manifestations of periodic fever, aphthous stomatitis, pharyngitis, and adenitis (PFAPA). Single-dose corticosteroids were effective in the abrupt cessation of acute attacks. Systemic juvenile idiopathic arthritis (s-JIA) was suspected in P6 at the age of 3.6 years based on the presence of fever, rash, lymphadenectasis, hepatosplenomegaly, polyarthritis and a positive (1:100) titer of antinuclear antibody (ANA). Although she received prednisone, diclofenac and naproxen treatment, recurrent fever and joint swelling were still intermittent. Unfortunately, she died of systemic inflammatory response syndrome at the age of 4.5 years.

### Clinical manifestations of patients with central fever

**Fig. 2:**
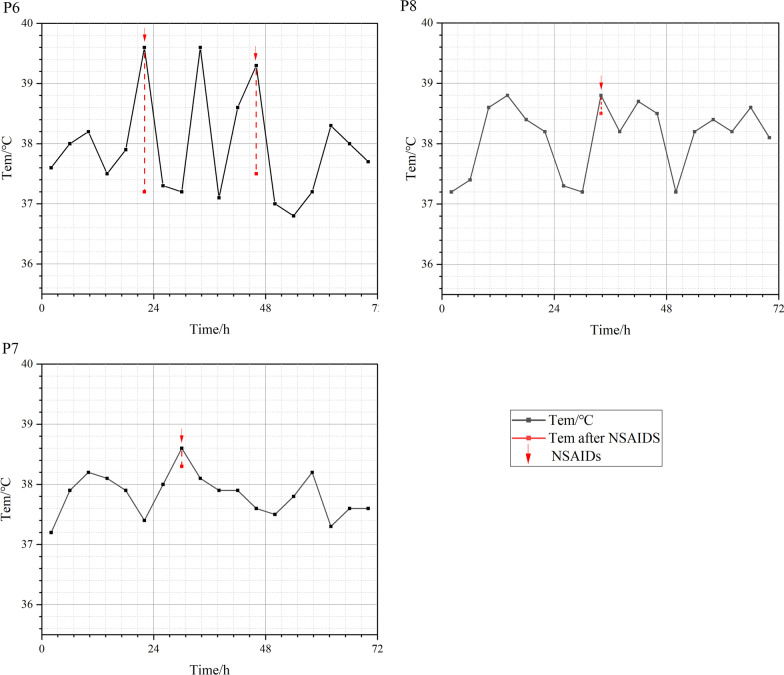
Temperature fluctuation and drug response to NSAIDs in some patients. P6: inflammatory fever, P7 and P8: central fever

Two patients (P7 and P8) presented with persistent fever attacks with a fluctuating body temperature of approximately 1.5 °C (P7 between 37.2–38.6 °C and P8 between 37.2–38.8 °C). Fever developed beginning at the age of 6–9 months and was not apparently associated with any infectious or inflammatory manifestation. Acute phase reactants remained normal. A transient respiratory syncytial virus, *Haemophilus influenzae* and *Staphylococcus aureus* lung infection occurred in P7, while cytomegalovirus-DNA was detected in peripheral blood and *Clostridium difficile* toxin was positive in stool samples from P8; however, adequate anti-infection therapy was administered without any efficacy against the fever. Both patients had facial and congenital abnormalities, for example, P7 presented with cryptorchidism, macrocephaly and frontal bossing, and P8 presented with congenital aural atresia (CAA), ocular hypertelorism, low-set ears and high arched palate. They also presented with growth retardation and developmental delay. The Ages & Stages Questionnaire, Third Edition (ASQ-3 scale) score of P7 showed a suspected overall developmental delay. Epileptic seizures, myelinated dysplasia and ventriculomegaly were further separately confirmed by electroencephalogram examination and head magnetic resonance imaging (MRI). The ASQ-3 score of P8 showed abnormalities in the gross motor and fine motor domains. His sleep electroencephalogram revealed sharp waves, and MRI revealed small softening foci in the left centrum semiovale, although no visible convulsions occurred. Nonsteroidal anti-inflammatory drug (NSAID) treatments, such as ibuprofen and acetaminophen, did not exert any effect on fever in these patients. They were eventually diagnosed with central fever. The temperature changes in both patients are shown in Fig. 2.

### Abnormal chromosome structure and number

**Fig. 3:**
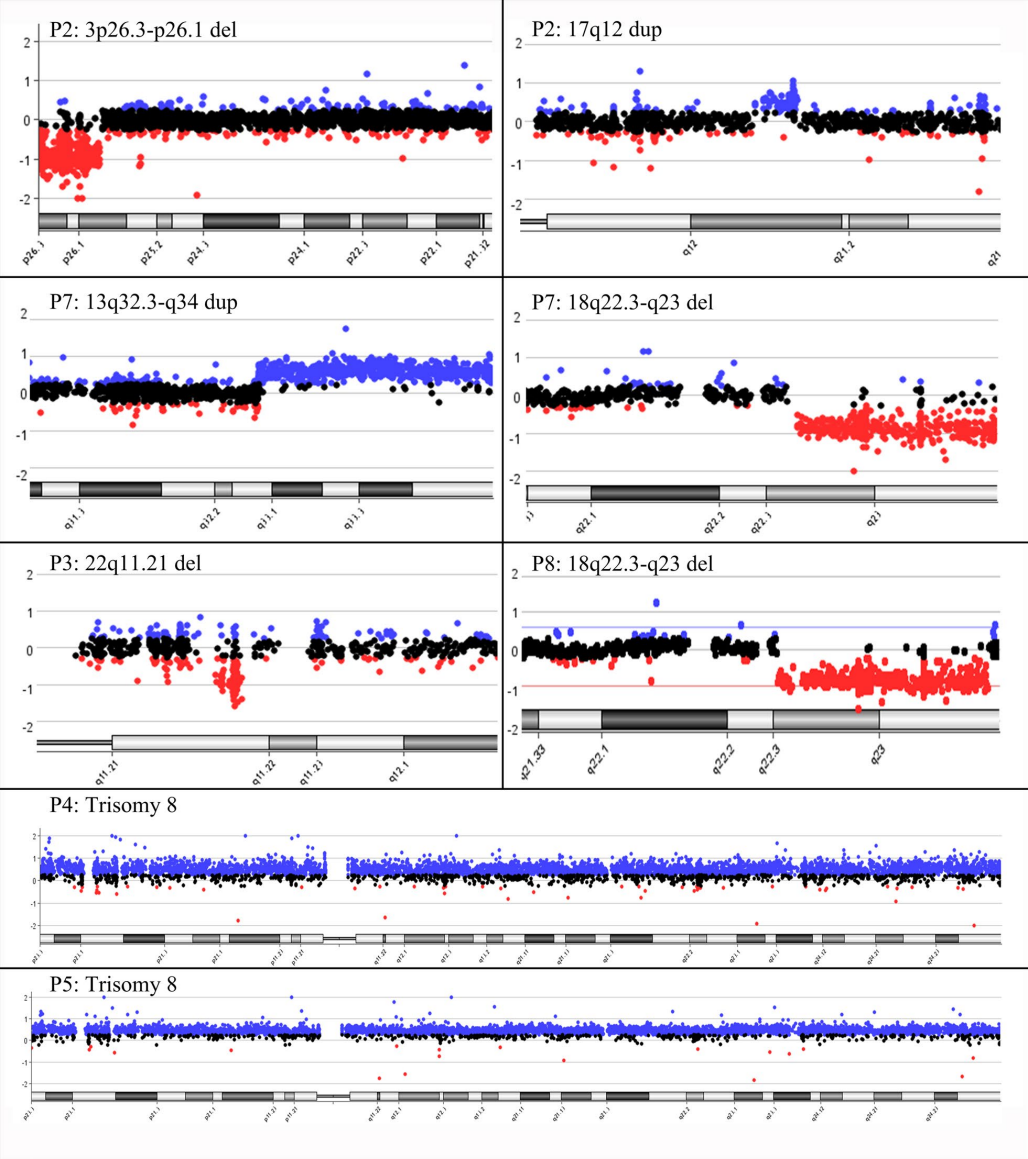
CNVs detected in patients with FUO by CGH in our cohort. 3p26.3-p26.1 del and 17q12 dup in P2; 13q32.3-q34 dup and 18q22.3-q23 del in P7; 22q11.21 del in P3; 18q22.3-q23 del in P8; trisomy 8 in P4 and P5, respectively

**Fig. 4:**
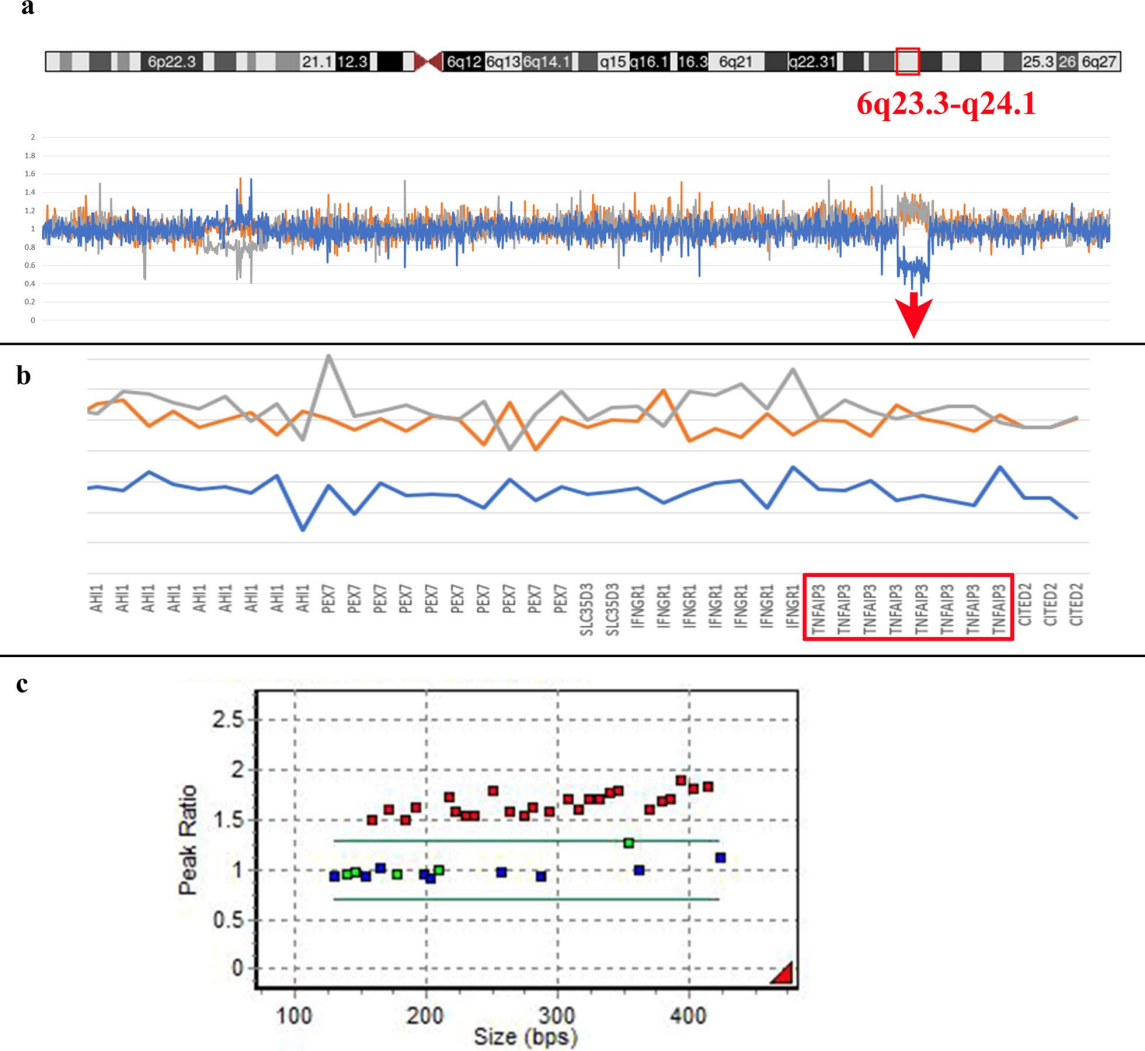
CNVs detected in P1 by MLPA and P6 by NGS. **a** A heterozygous deletion at 6q23.3-q24.1 was detected by the reduced NGS sequence reads in P6. Father (orange), mother (gray), P6 (blue); **b** *TNFAIP3* is included in the 6q23.3-q24.1 deletion region; **c** 7q11.23 dup in P1 by MLPA

CNVs were detected using trio-WES in these eight patients and verified with CGH (Fig. 3) or MLPA (Fig. 4c). Their parents had no FUO, and no chromosomal abnormalities were detected. In patients with inflammatory fever, we identified de novo CNVs: a 7q11.23 duplication in P1; a deletion at 3p26.3-p26.1 and a duplication at 17q12 in P2; a 0.7 Mb deletion at 22q11.21 (20,733,478–21,461,788) without involvement of the *TBX1* gene in P3; and a 3.576 Mb deletion at 6q23.3-q24.1 in P6 (Fig. 4a). The *TNFAIP3* gene was involved in the deleted fragment in P6 (Fig. 4b). Two patients (P4 and P5) were identified to have trisomy 8. In addition, P5 had another mutation, c.1508G > C, in *PTPN11*.

De novo 18q22.3-q23 del was identified in patients (P7 and P8) with central fever. Meanwhile, P7 also carried another CNV (13q32.3-q34 dup).

### Literature review

We searched PubMed using the search criteria "Chromosome Disorders" [MeSH Terms] OR "Copy number variation" OR "chromosome abnormality" OR "chromosome deletion" OR "chromosome duplication" OR " trisomy" AND "fever"[All Fields] AND "(humans)". Ultimately, 19 patients with FUO were found to have chromosomal abnormalities or CNVs, which might explain or partially explain the cause of fever [9, 18–31]. The data are summarized in Table 3.

**Table 3:** FUO related chromosomal abnormalities reported in the literature

Patients with chromosomal abnormalities	Classification of fever	Fever features	Age at fever	Sex	Concomitant symptoms	Inflammatory biomarkers (during fever)	Treatment	Congenital anomalies	Growth retardation	Developmental delays	Others	Refs.
6q23.2-q24.3 del	Inflammatory fever	recurrent fever, several FUO during first year	< 1y	M	Neutrophilic dermatosis, oral aphthae, diarrhoea and perianal ulcers	Increased CRP, ESR	Methylprednisolone, etanercept	–	+	+	inverted CD4/CD8 ratio, ANA: 1:160 +	[18]
Trisomy 8	Inflammatory fever	recurrent fever, high fever	69y	M	Sweet's sydrome, MDS(RA), convulsion	Increased neutrophils, CRP, IL-6, G-CSF	NSAIDS, prednisone	–	NA	NA	–	[19]
	Inflammatory fever	periodic fever, for 2 years	1.5y	F	Chronic diarrhea, skin lesion, hydropericardium, MDS	Increased SolIL2R	Dexamethasone, colchicine	–	NA	NA	PTPN11 mutation	[20]
	Inflammatory fever	periodic fever, high fever	53y	F	Intestinal Behcet's-like disease, MDS	–	Colchicine, Azacitidine, prednisolone	–	NA	NA	MEFV E148Q mutation	[21]
	Inflammatory fever	periodic fever, for 1 year	66y	M	GIBD, macrocytic anemia	Increased WBC	Colchicine, mesalamine	–	NA	NA	–	[22]
	Inflammatory fever	periodic fever, high fever	72y	M	Erythema nodosum, MDS(RA)	Increased WBC, CRP	Colchicine, prednisolone	–	NA	NA	–	[23]
	Inflammatory fever	periodic fever, high fever	68y	M	Erythema nodosum, rhabdomyolysis and acute renal failure, diarrhea, MDS(RA), oral ulcer	Increased WBC, CRP	prednisolone	–	NA	NA	–	
15q del (maternal)	Central fever	persistent fever, 37.8–39.0 °C	1 m	F	-	–	No response to antibiotics and antipyretic medications	Facial anomalies	–	+	EEG: epileptiform discharges, seizures, microcephaly, hypotonia	[24]
	Central fever	persistent fever, 37.5- 39.0 °C	2 m	M	-	–	No response to antibiotics and antipyretic medications	Facial anomalies	–	+	EEG: epileptiform discharges, microcephaly, hypotonia, spastic tetraplegia	
15q del (paternal)	Central fever	persistent fever, 38- 39.0 °C	13y	M	Hypotension, hyperpyrexia, rhabdomyolysis, acute kidney injury	–	External cooling	Hypospadias	–	NA	obesity, obstructive sleep apnoea, asthma, hypertension, fatty liver disease, diabetes	[25]
	Central fever	persistent fever, 37.2–39.6 °C	15d	M	-	–	No response to antibiotics medications	Facial anomalies	–	NA	hypotonia, feeding difficulty	[26]
	Central fever	persistent fever, 37.6 -39.6 °C	6 m	M	-	–	NA	Facial anomalies	–	NA	hypotonia, hypogonadia	
	Central fever	persistent fever, 38.5- 39.5 °C	7 m	F	-	–	NA	Facial anomalies	–	+	Microcephaly, hypotonia	
	Central fever	persistent fever, high fever	Neonatal period	F	Rhabdomyolysis, MODS	–	Fluid resuscitation	Facial anomalies	NA	NA	deep venous thrombosis, seizure, hypotonia	[27]
16p13.3 del	Inflammatory fever	recurrent fever, 2–5/year	18y	M	Abdominal pain, arthritis	Increased CRP, ESR	Colchicine	Facial anomalies	+	+	MEFV M694V mutation	[28]
18q22.3-q23 del	Central fever	long-term fever, up to 40 °C	5 m	M	Hyporexia, poor weight gain	–	No response to antibiotics and antipyretic medications	Facial anomalies, congenital aural atresia, congenital vertical talus	+	+	delayed myelination, hypotonia	[29]
18q21.32-q22.2 dup; 18q22.2-qter del	Inflammatory fever	recurrent fever, frequency > 1/month	3w	M	Rash, abdominal pain, lymphadenopathy, constipation	NA	NA	Facial anomalies, cleft palate, TOF	NA	+	–	[9]
19q13.42 dup	Inflammatory fever	recurrent fever associated with s-JIA, spiking fever	2y	F	s-JIA	NA	Refractory to conventional treatment, Tocilizumab	–	NA	NA	–	[30]
22q13.33 del	Undetermined	irregularly fever, 37–38 °C, approximately 1/month	23y	F	Acute and transient psychotic disorder	NA	Olanzapine, valproic acid, haloperidol, oxazepam	Facial anomalies	–	+	–	[31]

*m* months; *y* years; *d* days; *w* weeks; *F* female; *M* male; *NA* not available; *FUO* fever of unknown origin; *CRP* C-reactive protein; *ESR* erythrocyte sedimentation rate; *ANA* anti-nuclear antibodies; *MDS* myelodysplastic syndrome; *RA* refractory anemia; *IL-6* interleukin-6; *G-CSF* granulocyte colony-stimulating factor; *WBC* white blood cells; *NSAIDs* non-steroidal anti-inflammatory drugs; *SolIL2R*: soluble IL-2 receptor; *GIBD* gastrointestinal Behcet's disease; *EEG* electroencephalo-graph; *MODS* multiple organ dysfunction syndrome; *TOF* tetralogy of fallot*; s-JIA* systemic onset juvenile idiopathic arthritis

In previous reports, trisomy 8 was detected most frequently in inflammatory fever, often leading to periodic fever and Behcet’s-like disease similar to P4 and P5 in our study. Patients with 6q23.2-q24.3 del, 16p13.3 del, chromosome 18 rearrangement (18q21.3-q22.2 dup/18q22.2-qter del) and 19q13.42 dup were described as having recurrent fever, which was considered systemic inflammatory diseases, for example, s-JIA. Seven patients with 15q del (2 maternal and 5 paternal) and one patient with 18q22.3-q23 del were reported to have central fever. Central fever was usually accompanied by facial anomalies and neurological involvement, including developmental delay, microcephaly, hypotonia, and epileptiform discharges. Additionally, one patient reported to have 22q13.33 del suffered from an acute and transient psychotic disorder accompanied by irregular fever, which was not classified as inflammatory fever or central fever.

**Fig. 5:**
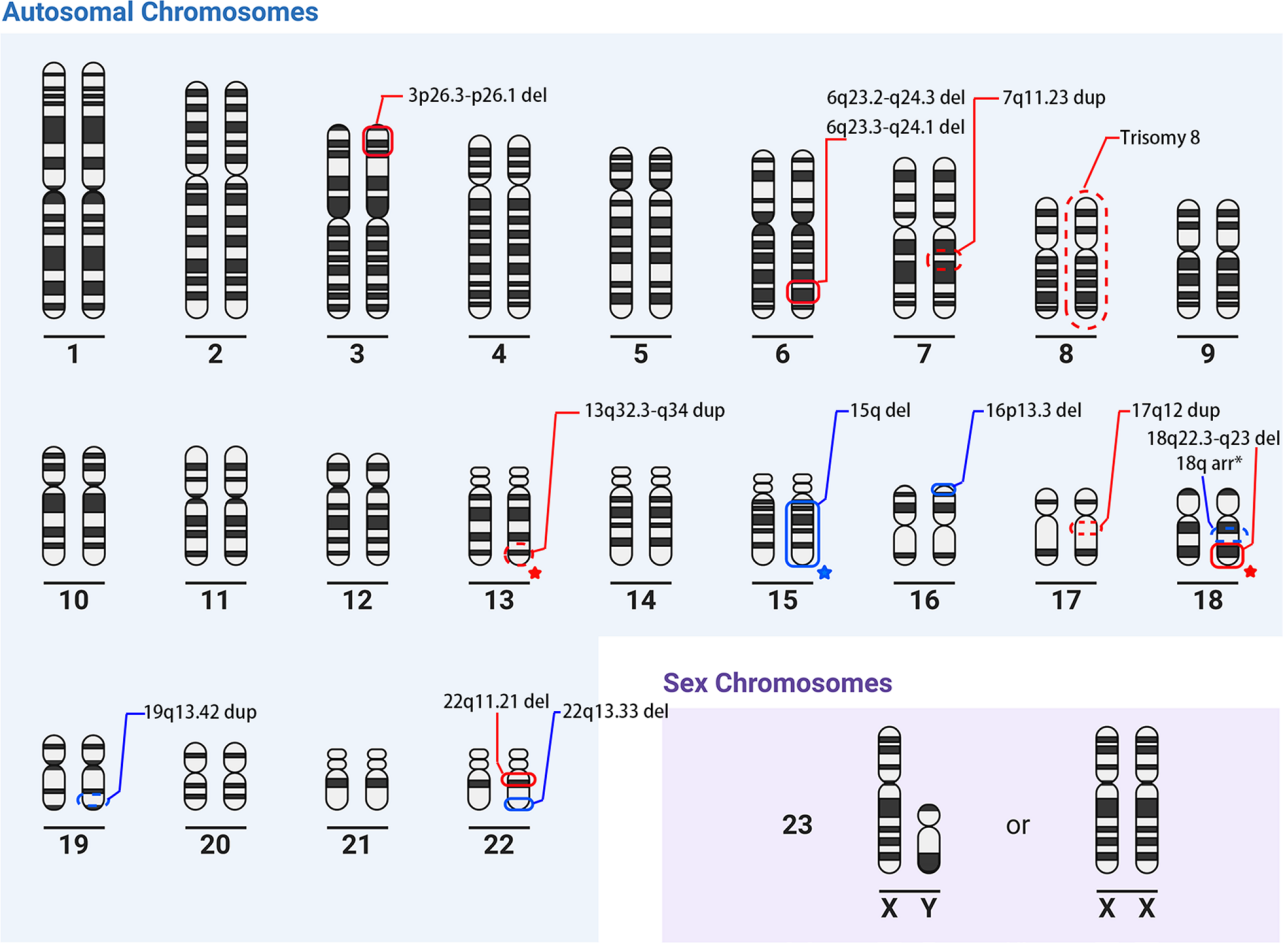
Chromosome map related to FUO. The fragments detected in our cohort are shown in red, and those reported before are shown in blue. The solid and dashed lines denote deletion and duplication, respectively. Trisomy 8, 6q23.3-24.1 del and 18q22.3-q23 del have been both involved in our and previous studies. (18q arr*: 18q rearrangement)

In addition to trisomy 8, 6q23.3-q24.1 del and 18q22.3-18q23 del, our study also revealed additional CNVs that may be related to FUO, such as 7q11.23 dup, 3p26.3–26.1 del/17q12 dup, and 22q11.21 del. A chromosome map related to FUO was drawn (Fig. 5).

## Discussion

FUO is a challenging disease. Recent studies have shown the importance of performing NGS in adult patients with FUO [32]. However, limited genetic data are available in pediatric patients [33]. In our study, 145 patients presenting with FUO during the study period were enrolled, and 24.1% (35/145) of these patients were diagnosed with monogenic autoinflammatory diseases. However, some cryopyrin-associated periodic syndromes caused by low-frequency somatic *NLRP3* variants, especially the p. E567K variant, may have been missed by WES due to the depth of coverage [34]. Moreover, the detection of chromosomal abnormalities in patients with FUO was 5.5%, which should not be ignored. No previous reports have focused on the overall proportion of chromosomal abnormalities in patients with FUO. The proportion of chromosomal abnormalities in our FUO cohort was significantly higher than that in the general pediatric population, ranging from 0.43% to 0.83% in live births [35–37], and there were also chromosomal spectrum differences, suggesting the significance of chromosomal-related tests in FUO.

Recently, several studies have begun to focus on chromosomal abnormalities leading to inflammatory fever. Hiromi Tadaki et al. reported recurrent fever in a patient with s-JIA who carried 19q13.42 dup, which contains the NLRP family associated with the inflammatory pathway [30]. Chromosomal rearrangement (18q21.3-q22.2 dup/18q22.2-qter del) encompassing a duplication of *TNFRSF11A* has been reported to be associated with recurrent fever, rash and lymphadenopathy [9]. CNV detection approaches are recommended as routine diagnostics in patients with systemic autoinflammatory diseases for whom a confirmatory genotype is unavailable [10, 18]. Chromosomal imbalances involving either the loss or gain of large genomic regions may be detectable with molecular cytogenetic techniques such as CGH, and smaller CNVs can also be detected by MLPA [10].

The discovery of chromosomal abnormalities in patients with FUO can guide diagnosis and treatment for better management strategies. In our cohort, six patients with inflammatory fever had chromosomal abnormalities, two of which were trisomy 8. Both patients with constitutional trisomy 8 [22, 38, 39] and patients with trisomy 8 in their bone marrow [19–21, 23, 40, 41] have been reported to exhibit Behcet’s disease (BD) or other inflammatory disorders. CRP and interleukin (IL)-6 levels were significantly increased in patients with trisomy 8 during acute inflammation [20, 42]. Inflammatory manifestations related to trisomy 8 in children are rarely reported. Two patients in our study diagnosed with trisomy 8 had symptoms similar to BD, along with increased CRP/ESR levels and elevated IL-6 levels. Treatment with anti-inflammatory medications, such as systemic steroids, can improve clinical symptoms [20, 39]. Trisomy 8 is also thought to increase the risk of the development of malignancy, especially myelodysplastic syndrome (MDS) symptoms [40, 43]. Associations between intestinal BD, MDS and trisomy 8 have been described. In addition, P5 also carried the gene mutation c.1508G > C in *PTPN11*. *PTPN11* encodes the protein tyrosine phosphatase SHP2, a signal protein involved in the RAS/MAPK pathway [20]. The mutation c.1508G > C, either germline or somatic, has been reported to be associated with juvenile myelomonocytic leukemia [44, 45], which requires hematopoietic stem cell transplantation.

*TNFAIP3* is located in the 3.576 Mb deletion region on chromosome 6q23.3-q24.1, which was identified in P6. *TNFAIP3* mutation may cause insufficient production of A20, which finally results in negative feedback inhibition of the NF-κB signaling pathway. This mutation has been reported to be the genetic factor underlying many autoinflammatory and autoimmune diseases, such as BD and systemic lupus erythematosus [46, 47]. One patient with 6q23.2-q24.3 del was previously described to have a systemic autoinflammatory disease characterized by recurrent fever, oral aphthae and perianal ulcers [18]. P6 clinically manifested with early-onset s-JIA and showed a poor response to corticosteroids and NSAIDs. Cytokine inhibitors such as tumor necrosis factor (TNF) inhibitors can be further considered to inhibit inflammatory responses [47]. Loss of the tumor suppressor gene *TNFAIP3* may lead to B-cell lymphomas [48, 49]. Deletions in 6q23.3-6q24.1 have recently been identified by aCGH in ∼30% of primary mediastinal B-cell lymphomas [48]. Long-term attention should be given to the occurrence and development of tumors in patients with chromosomal abnormalities.

Furthermore, some CNVs in our study have not been previously reported to be related to inflammatory fever, such as a duplication at 7q11.23 in P1, a deletion of 3p26.3-p26.1 and a duplication at 17q12 in P2, and a 0.7 Mb deletion of 22q11.21 in P3. Common CNVs in four chromosomal “hot spots” can result in deletions or duplications in the 22q11.2, 7q11.23, 17p11.2, and 16p11.2 regions [8]. The population frequency of 7q11.23 duplication is relatively high (1:7,500–20,000), and children can mainly present with developmental delays/intellectual disabilities and behavioral problems, which is consistent with P1 [50]. Moreover, P1 was hospitalized repeatedly due to fever and tonsillitis. In addition to autoinflammatory symptoms that may be consistent with PFAPA, P3 experienced growth retardation and developmental delay, which are considered exclusion criteria for PFAPA, according to the modified Marshall's criteria [51]. A central 22q11.2 deletion was detected in P3, which was relatively rare compared to the typical 3 Mb 22q11.2 deleted region. The central 22q11.2 deletion can result in developmental problems [52], such as developmental delay. It is possible that those high-frequency chromosomal abnormalities are found incidentally in FUO. Subsequent case reports are required to clarify the potential association between CNVs detected and fever.

No clear statistics are available on the association between central fever and genetic factors. Deletion of the long arm of chr18 was previously described as a common autosomal syndrome with an incidence of 1:40,000 live births [29]. Patients with this syndrome are characterized by short stature, facial dysmorphism, foot deformities, CAA, variable intellectual disability and neurological abnormalities [53]. Neurological abnormalities may include hypotonia, seizures, nystagmus, poor coordination, tremor and abnormal signals on brain MRI. After a literature review, we identified one patient who presented with thermic dysregulation with one of the smallest 18q22.3-q23 interstitial deletions described thus far [29]. Two more patients (P7 and P8) with fever and 18q deletion syndrome analyzed in our study indicated that thermal dysregulation may be a symptom related to this disease rather than an occasional finding. Deficiency or dysfunction of the involved gene, *GALR1*, which encodes the galanin receptor GALR1 and mediates the hyperpolarization of warm sensitive neurons in the preoptic area of the hypothalamus, was speculated to be the cause of fever. In addition, P7 carried a duplication at 13q32.3-q34, which was associated with congenital malformation of the brain, kidney, limb and lung in previous reports [54]. Overall, most patients (6/8) in our cohort had developmental delay, highlighting the need for chromosomal testing in children with FUO and developmental delay. Long-term rehabilitation training is necessary for these patients.

## Conclusions

In conclusion, we summarized the genetic characteristics of children with FUO at a single Chinese center and emphasized the role of chromosomal abnormalities in FUO. The characteristics of chromosomal abnormalities leading to fever were systematically summarized. Our study revealed several chromosomal abnormalities known or potentially related to central and inflammatory fever. We suggest that CNV detection approaches should be routinely performed in patients with FUO, especially in patients with obvious signs of systemic inflammatory manifestations or developmental delay, which may reveal the genetic basis of FUO in these patients. Identifying chromosomal abnormalities may change the diagnosis and care of these patients.

## Data Availability

The datasets used or analyzed during the current study are all included within the article and are available from the corresponding author upon reasonable request.

## Abbreviations

AML: Acute myeloid leukemia
a-CGH: Array comparative genomic hybridization
ANA: Antinuclear antibody
CAA: Congenital aural atresia
CNV: Copy number variation
CRP: C-reactive protein
BD: Behcet’s disease
ESR: Erythrocyte sedimentation rate
FUO: Fever of unknown origin
HLH: Hemophagocytic lymphohistiocytosis
IL: Interleukin
MDS: Myelodysplastic syndrome
MLPA: Multiplex ligation-dependent probe amplification
MRI: Magnetic resonance imaging
NGS: Next-generation sequencing
NSAIDs: Nonsteroidal anti-inflammatory drugs
PFAPA: Periodic fever, aphthous stomatitis, pharyngitis, and adenitis
SAA: Serum amyloid A
s-JIA: Systemic juvenile idiopathic arthritis
TNF: Tumor necrosis factor
WBC: White blood cell count
WES: Whole exome sequencing
